# High-Quality Draft Genome Sequence of *Lactobacillus casei* Strain Z11, Isolated from a Human Adult Intestinal Biopsy Sample

**DOI:** 10.1128/genomeA.00634-17

**Published:** 2017-07-13

**Authors:** Martín S. Marcial-Coba, Ian P. G. Marshall, Lars Schreiber, Dennis S. Nielsen

**Affiliations:** a Food Microbiology Section, Department of Food Science, University of Copenhagen, Copenhagen, Denmark; b Department of Bioscience, Center for Geomicrobiology, Aarhus University, Aarhus, Denmark

## Abstract

Several *Lactobacillus casei* strains are used as probiotics. *L. casei* strain Z11, isolated from a human colon biopsy sample, has been suggested as a probiotic candidate based on promising properties *in vitro*. Here, we present a 2.74-Mbp high-quality draft genome sequence for this strain.

## GENOME ANNOUNCEMENT

*Lactobacillus casei* is widely distributed in a large number of habitats, being isolated from multiple food products (1). Since it is genetically well adapted to the gastrointestinal and reproductive tract environment of humans and animals (2), several *L. casei* strains are used as probiotics in functional foods (3, 4).

Some beneficial effects of probiotics are associated with short-chain fatty acid production (5), mainly of butyrate (6), which is consumed by intestinal epithelial cells (IECs), increasing mucin production and improving gut barrier function (7).

Adhesion to IECs is another desirable characteristic of probiotic strains. In that context, sortases catalyze surface protein anchorage to the bacterial cell wall (8), playing key roles in bacterium-host interactions (9).

Larsen et al. (10) showed that* Lactobacillus casei* Z11 exhibits a moderate binding ability to the porcine epithelial cell line IPEC J2. Furthermore, Weiss et al. (11) showed a strong immune stimulatory capacity of this strain when added *in vitro* to dendritic cells. These findings suggest that *Lactobacillus casei* Z11 can be considered a probiotic candidate after further investigation.

Full-genome sequencing of *Lactobacillus casei* Z11 is required to generate a genomic basis in order to reinforce the probiotic potential of this strain.

Genomic DNA was extracted and purified using a GenElute bacterial genomic DNA kit (Sigma-Aldrich, Germany). A sequencing library was prepared using the Nextera XT kit (Illumina, USA). Genome sequencing was performed using the Illumina MiSeq platform, with a paired-end 300-bp MiSeq reagent kit version 3. The resulting sequence reads (ca. 4.55 million read pairs; ~1.2 Gbp) were inspected for data quality using FastQC version 0.11.5 (http://www.bioinformatics.babraham.ac.uk/projects/fastqc). Reads were trimmed using Trimmomatic version 0.36 (12) with the following parameters: CROP, 290; HEADCROP, 19; SLIDINGWINDOW, 4:20; and MINLEN, 100. Trimmed reads (ca. 3.7 million read pairs; ~0.8 Gbp) were assembled using SPAdes version 3.9 (13) with the following parameters: -k 21,33,55,77,99,127 –careful. Genome coverage of assembly contigs and G+C content were determined using BBMap version 35.x (https://sourceforge.net/projects/bbmap/). Scaffolds were filtered based on the following parameters: G+C content, 35% to 55% retained; coverage, 0.21- to 3,900-fold retained; and minimum length, 150 bp.

The decontaminated high-quality draft genome of *Lactobacillus casei* Z11 has a total length of 2,744,915 bp in 193 scaffolds, an average G+C content of 46.4%, and an *N*_50_ length of 38,635 bp. Using the gene marker set for the species *Lactobacillus casei*, based on 16 genomes, CheckM version 1.0.6 (14), the genome was estimated to be 99.5% complete.

In total, 2,617 coding sequences, 6 genes encoding rRNA (including 4 5S, 1 16S, and 1 23S), 55 tRNAs, and 1 transfer-messenger RNA (tmRNA) were determined by Prokka version 1.12-beta (15).

The presence of the butyrate kinase gene *buk*, involved in butyrate synthesis, reinforces the probiotic potential of this strain; also, *srtA* and *srtC*, encoding sortase A and C, respectively, might play a significant role in the adhesion to IECs.

### Accession number(s).

The *Lactobacillus casei* Z11 genome sequence has been deposited at DDBJ/ENA/GenBank under the accession number MPOP00000000. The version described in this paper is version MPOP01000000.
